# Benzos (as) needed: research into as-needed and intermittent benzodiazepines for anxiety is required for comprehensive best prescribing practices

**DOI:** 10.3389/fpsyt.2025.1569416

**Published:** 2025-04-28

**Authors:** Arthur Krieger

**Affiliations:** Department of Philosophy, Temple University, Philadelphia, PA, United States

**Keywords:** benzodiazepines, anxiety disorder, pharmacotherapy, best practices, prescription drug safety, misprescription, as-needed, PRN

## Abstract

The medical and public health communities are divided around the use of benzodiazepine (“benzo”) pharmacotherapy for anxiety disorders. Recent years have seen increased attention to benzo overprescription and its risks, leading to a pervasive emphasis on deprescribing. Some have resisted this trend, arguing that the balance of evidence supports the safety and efficacy of benzo pharmacotherapy for both short-term and long-term treatment of anxiety disorders. Given that rising rates of anxiety disorders and benzo misuse are both serious public health concerns, there is an urgent need for comprehensive evidence-based best practices for the prescription of benzos for anxiety. At present, however, major scientific gaps make it impossible to formulate such guidance. Most concerning is the lack of research into as-needed and intermittent prescription and use, which are both what benzos are best suited for, and likely, how they are most commonly administered. Further research into the safety and efficacy of both long-term daily and intermittent, as-needed benzo prescription and use are badly needed. But a roundly “anti-benzo” sentiment may be causing problematic underprescription of benzos, particularly when superior alternatives like cognitive-behavioral therapy are not widely available.

## 1 Introduction

Benzodiazepines (“BZDs,” “BDZs,” “BZs,” or “benzos”) have been an essential pharmaceutical treatment for anxiety disorders since their introduction in the 1960s (1). Dependence was quickly recognized as a risk of regular benzo use, and as such, the safety and efficacy of benzos for anxiety have long been matters of interest (2, 3). Recent years have seen renewed controversy over the prescription of benzos that reveals widely divergent assessments of the drug class. While the safety and efficacy of short-term benzo use for anxiety is mostly settled (4–9), long-term benzos for anxiety—defined as daily use for more than one month—remains a subject of deep disagreement. On one hand, some authorities view long-term benzo use as carrying unacceptable risks of dependence, overdose and death, in addition to other adverse effects like cognitive impairment (10–12). National and international public health institutions have come to emphasize the avoidance of long-term benzo prescription in recent years, citing a high risk of dependence in particular (e.g. 13–18; see also, 19). Perceived overprescription of benzos for long-term use has led to intense interest in deprescribing, with the aim of tapering long-term benzo users *en masse* (20–25). As Modesto-Lowe and colleagues put it, “Given the harms of long-term use, deprescribing BZDs has become the ‘mantra’ in clinical discourse” (24, 205). On the other hand, however, many authorities argue that even long-term benzo use is a safe and effective treatment for various anxiety disorders, sometimes framing increased caution around benzo prescribing as alarmism (26–30). Soumerai and colleagues tell us that “Weak science, alarming FDA black box warnings, and media reporting have fueled an anti-benzodiazepine movement that at times even portrays appropriate BZRA [i.e. benzo and z-drug] prescribing as a gateway to long-term dose escalation, tolerance, and drug misuse” (30, 186). What members from each camp agree on is the need for evidence-based best practices for the prescription of benzos. While recent contributions to the debate around benzo prescribing are steps in the right direction, consensus lies far in the distance. Much more research is needed to settle the debate, research for which there are few financial incentives (26, 319).

Several crucial yet unacknowledged gaps in benzo research concern as-needed or *pro re nata* (PRN) prescription and use outside the hospital setting. As-needed prescribing to psychiatric inpatients has received some attention, especially as a site for deprescribing (31–36). But there has been little research on how medical providers prescribe benzos to anxiety patients for as-needed use in daily life; how anxiety patients use the benzos they are prescribed for as-needed use; and the safety or efficacy of non-daily, intermittent benzo use (cf. 37–40). This broad scientific blind spot is problematic for several reasons. As fast-acting anxiolytics, benzos are particularly well-suited to the treatment of unexpected acute anxiety, such as often occurs in specific phobias, social anxiety disorder, and panic disorder. Crucially, there are few pharmaceutical alternatives for acute anxiety, and none that are equally safe, effective, and well-studied. Given their suitability for this application, benzos are often prescribed for as-needed use, i.e. for when acute anxiety unexpectedly arises. Indeed, this is likely how they are most commonly prescribed (37, 41), meaning there are probably many millions of people in the U.S. alone who are prescribed benzos as-needed for anxiety (41–43). Furthermore, rising caution around benzos and the deprescription movement are taking place in the midst of a mental health crisis in the U.S. and elsewhere characterized by rising rates of anxiety disorders (44–46), and reduced access to preferred non-pharmaceutical alternatives to benzos like cognitive-behavioral therapy (CBT) (47–50). There is therefore a serious risk that many patients who do or would benefit from as-needed benzo prescriptions will lose access to them without having any satisfactory alternative. In short, then, more research into as-needed benzo prescribing is urgently needed, so that it is possible to formulate truly evidence-based best practices and to ensure that anxiety patients get the pharmaceutical treatment they need.

The aim of this paper is to highlight gaps in the research around the safety and efficacy of benzos for anxiety, with special attention to as-needed and intermittent prescription and use. Moreover, it will make suggestions concerning how to prescribe benzos for anxiety that reflect the best information currently available, given the nonideal scientific and public health circumstances. I will proceed as follows. In section 2, I will review some basic information about benzos. In section 3, I will summarize and assess the debate around long-term prescription and use for anxiety, including research gaps. In section 4, I will discuss key research gaps concerning as-needed and intermittent prescription and use of benzos for anxiety. In section 5, I will explain why comprehensive best practices for benzo anxiety treatment are so urgently needed. And in section 6, I will suggest some provisional best practices.

## 2 An overview of benzodiazepines

Benzos are central nervous system depressants noted for their anxiolytic (anxiety-reducing), hypnotic (sleep-inducing), and sedative (relaxing) effects. They are widely prescribed for anxiety and insomnia, as well as for other conditions including seizures, alcohol withdrawal, psychomotor agitation, muscle spasms, parasomnias, obsessive-compulsive disorder, and delirium. Well-known benzos include diazepam (“Valium”), alprazolam (“Xanax”), and lorazepam (“Ativan”). Benzos depress the central nervous system by enhancing the effects of the neurotransmitter gamma-aminobutyric acid A (GABA_A_) at GABA_A_ receptors throughout the brain. Another class of pharmaceuticals, referred to as “nonbenzodiazepines” or “z-drugs,” do not have the benzene-diazepine chemical structure of benzos but work on GABA_A_ receptors in the same way. One well-known z-drug is zolpidem (“Ambien”). Benzos and z-drugs are sometimes classed together as benzodiazepine-receptor agonists (BZRAs).

The benzodiazepine drug class is diverse. Benzos vary in how potent they are, and in how long their effects last. Some are highly potent with short half-lives, some are highly potent with long half-lives, some are milder with short half-lives, and some are milder with long half-lives. So different benzos suit different applications (51). But, in general, what distinguishes the benzo drug class is that, as depressants, they are relatively fast-acting and relatively potent. A person who is entering an anxiety attack can take a benzo and, within an hour, will be mostly or entirely de-escalated. A person whose racing thoughts keep them up at night will be asleep within an hour of taking the right BZRA.

Dependence is the primary danger of regular benzo use that cuts across all demographics. A person who takes a potent benzo once a day for as little as two weeks can experience a mild withdrawal upon stopping (52, 137; 53, 639). The higher the dose a person takes, the longer and more frequently they take it, and the more potent the drug, the more severe dependence can become (54, 55). Patients who have been prescribed high doses of benzos for many years on end have reported horrific and unusual withdrawal symptoms upon cessation that can last for months or even years (28, 56), and that can complicate tapering and deprescription (57). Along with alcohol and barbiturates, benzos are some of the only drugs whose withdrawals can be fatal. As a general statement it is probably safe to say that withdrawal from benzos is more dangerous, and more difficult, than withdrawal from opioids. Since severe withdrawal symptoms can negatively reinforce drug use (as a way of avoiding withdrawal), benzos have significant potential for misuse and can be considered addictive. For this reason, benzos are controlled substances in many jurisdictions. In the U.S., for instance, benzos are classed by the Controlled Substances Act as Schedule IV drugs, meaning that they have “low [but significant] potential for abuse and low [but significant] risk of dependence” (58). Benzos carry other risks, too, particularly for older patients. As depressants, they decrease motor coordination and increase the risk of car accidents and falls, with falls being a major concern for older patients (59, 60). There is debate around whether long-term benzo use increases the likelihood of cognitive problems and dementia, especially in older patients (61–63). Relatedly, there is conflicting data around whether long-term daily benzo use results in pathological changes to brain structures, and in particular, dilatation of the ventricular system. Some studies have found increased ventricle-brain ratio in long-term benzo users (64–66), while other studies found no significant differences from control groups (67–69).

While benzos carry certain risks, there are few pharmaceutical alternatives with comparable onset and efficacy for acute anxiety, and none that are as safe, effective, and well-studied. Drugs sometimes prescribed for chronic anxiety, like SSRIs, SNRIs, and buspirone, take weeks to bioaccumulate and become effective, and cannot be taken as-needed for acute symptoms. Beta blockers like propranolol can be taken as-needed for acute anxiety, but only address somatic manifestations (e.g. tremors, sweating, tachycardia). Historical alternatives such as barbiturates and meprobamate have been largely replaced by benzos, which are significantly safer. Perhaps the most promising pharmaceutical alternative to benzos for acute anxiety is hydroxyzine, a first-generation antihistamine that produces anxiolytic sedation within 15-30 minutes of administration. Unlike benzos, hydroxyzine has no significant potential for dependence or misuse and is not a controlled substance, which may make providers more comfortable prescribing it. But research on hydroxyzine for anxiety is limited, and available evidence suggests that there are many cases in which benzos are preferable. To start, research on hydroxyzine for anxiety is limited to safety and efficacy for generalized anxiety disorder (GAD); there is no safety or efficacy research for other anxiety disorders. A Cochrane review found that hydroxyzine appears to outperform placebo for GAD and is roughly as safe and effective as regularly dosed benzos for this indication. But the review also found that the literature on hydroxyzine for GAD is not sufficiently robust to sustain a decisive comparison with benzos (70). There is evidence that hydroxyzine is less effective for acute anxiety than the benzo alprazolam in comparable doses (71), and some authors who are concerned about benzo overprescription nevertheless express the worry about substituting “medications with less evidence of anxiolytic benefits, such as low-dose quetiapine [an antipsychotic not indicated for any anxiety disorders] or hydroxyzine” for benzos (27, 73). Tolerance to the sedative effects of hydroxyzine appears to develop in as little as seven days (72)—much more rapidly than tolerance to the anxiolytic effects of benzos—which may limit hydroxyzine’s utility for short-term use between one and four weeks. (It is not clear that the anxiolytic efficacy of hydroxyzine is separable from its sedative efficacy.) Also, hydroxyzine is more strongly hypnotic than most benzos (70, 2), making it less attractive for daytime use, and potentially conducive to automotive accidents and falls. In short, then, while hydroxyzine is likely a safer treatment for acute anxiety than benzos in some cases, neither its safety nor its efficacy for this application has been extensively studied.

One of the most directly comparable substances to benzos is often overlooked in the medical literature, presumably because it is not a prescription pharmaceutical: ethanol, or drinking alcohol. Like benzos, alcohol is a “GABAergic” depressant with rapid-onset anxiolytic effects. While alcohol is not an alternative to benzos in clinical contexts, in most jurisdictions it is an easily accessible means of self-medication for patients struggling with anxiety. There is evidence that patients who lose access to prescription benzos often turn to alcohol instead (73, 89; 74, 103-107; 2, 222). Crucially, alcohol is markedly more dangerous than benzos, both in the short term (as an intoxicant) and in the long term (as a habit-forming toxin and carcinogen) (75).

## 3 The debate over long-term benzodiazepine prescription and use

The first benzodiazepine, chlordiazepoxide (“Librium”), was introduced in 1960 and was quickly followed by several others. Benzos have been some of the most prescribed drugs in the world since at least the 1970s (1). The medical community quickly became aware that benzos are habit-forming, and there have been periods in which efforts to reduce dependence led to lower prescription rates in the U.S. and other countries (2, 219; 3). But benzos remain extremely popular in the 21st century, with rates of prescription increasing between the mid-1990s and the mid-2010s (41–43).

The current wave of heightened attention to the dangers of benzos is largely due to their increasing role in fatal drug overdoses—and the apparent role of medicine in feeding this problem. According to one widely cited study, the number of adults in the U.S. filling a benzo prescription increased 67 percent (from 8.1 million to 13.5 million) between 1996 and 2013, and the total quantity of diazepam equivalents in benzos more than tripled from 1.1 to 3.6 kilograms per 100,000 adults. Meanwhile, overdose deaths involving benzos increased more than five-fold in the same time period, from 0.58 deaths per 100,000 adults to 3.07 (43, 686-687). Benzo-related overdose deaths are rarely due to benzos alone, but rather, to the mixing of benzos with other depressants like opioids and alcohol. The co-prescription of benzos and opioids seems to be a significant iatrogenic source of fatal overdose. The co-prescription of benzos and opioids went up dramatically in recent years, along with overall benzo-related overdose deaths (41). Furthermore, reductions in overall opioid prescribing helps to explain the flattening of benzo-related overdose death rates in White, non-elderly users starting in 2010 (43, 688). It is the putative role of benzo misprescription in feeding a larger opioid-related overdose crisis that led the FDA to issue its first of two “boxed warnings” for benzos, to be included on all prescription benzo labels, in 2016 (then called “black box warnings”). Boxed warnings are the FDA’s highest warning about medication safety, and the 2016 boxed warning cautioned prescribers and patients specifically about the risk of overdose and death in mixing benzos and opioids [U.S. Food and Drug Administration, (15)].

New attention to benzo-related overdose risk appears to have informed the larger discourse around benzo prescribing. In a 2018 article for the *New England Journal of Medicine*, leading addiction researcher Anna Lembke and colleagues start from the acknowledged danger of opioid-benzo coprescription, but quickly turn to highlight other dangers of long-term benzo use. “Many prescribers,” they claim, “don’t realize that benzodiazepines can be addictive and when taken daily can worsen anxiety, contribute to persistent insomnia, and cause death” (10, 694). Furthermore, they suggest that just as patients sometimes turn to street opioids when they have become dependent but can no longer obtain a prescription, patients who become dependent on prescription benzos may turn to stronger, “designer” street benzos like clonazolam as an alternative to pharmaceuticals (693-694; see also, 76). For these authors, the upshot is that there is a need for “the reduction of overprescription” through improved medical education and the use of alternatives to long-term benzo prescription whenever possible (694). Lembke and colleagues’ emphasis on the manifold dangers of long-term benzo use appears in the second and latest FDA boxed warning for benzos, which asserts that benzos carry “serious risks of abuse, addiction, physical dependence, and [potentially deadly] withdrawal reactions,” even when they are taken as prescribed, and without co-prescription of opioids [U.S. Food and Drug Administration, (16)]. A general precautionary tone is also detectable in other recent treatments of benzo epidemiology (77–79) and prescription practices (16). There is growing consensus in the medical community and among public health institutions that benzos are simply not a first-line treatment for anxiety (13, 19) with some authors going so far as to say that they are not meant for long-term use at all (80, 4; 12).

The last couple years have seen significant expert resistance to putative alarmism around benzo overprescription (26–30). According to these authors, benzos are relatively safe and effective for at least some anxiety disorders (e.g. panic disorder, GAD), some cases of insomnia, and other medical conditions, including for long-term use. Stephen Soumerai and colleagues announce that “Weak science, alarming FDA black box warnings, and media reporting have fueled an anti-benzodiazepine movement that at times even portrays appropriate BZRA prescribing as a gateway to long-term dose escalation, tolerance, and drug misuse” (30, 186). Indeed, available evidence suggests that patients rarely misuse benzo prescriptions, rarely require dose escalation to maintain anxiolytic efficacy, and rarely want to discontinue benzo therapy due to adverse effects—all of which are inconsistent with high risk of severe dependence or addiction. A cross-sectional analysis of 2015 and 2016 National Survey on Drug Use and Health data finds that about 18 percent of benzo use in the U.S. is misuse, i.e. does not follow medical guidance; that the most common kind of misuse, is use without any prescription at all (as opposed to misuse of prescribed benzos); and that young age and opioid misuse are the characteristics most strongly associated with benzo misuse (81; see also, 79). Misuse of prescribed benzos is therefore very low across most or all demographics. With regards to dose escalation in long-term use, the largest study ever on the topic (N=950,767) finds that only 15 percent of Danish BZRA prescribees used them for at least one year; that only 7 percent escalated to doses above the recommended level; and that there is no indication of dose escalation among BZRA users of at least three years (82). These results confirm findings of earlier studies, which also found very low rates of multi-year use or dose escalation (83, 84). Regarding patient-driven discontinuation, a review of the records of one general psychiatric practice found that only seven out of 836 benzo prescribees (0.8 percent) discontinued benzos due to adverse events. Furthermore, all of these adverse events were related to the abuse of other drugs, and none resulted in serious injury or death (85). Medical and public health experts increasingly believe that, even if benzos are effective for certain indications, their risks are at least greater than those of the pharmaceutical alternatives, like SSRI and SNRI antidepressants. But the most comprehensive literature review on safety and efficacy of benzos across all indications finds that for several conditions and some anxiety disorders, they are the best tolerated and most effective pharmaceutical intervention (26, 318; see also, 86, 87). These authors therefore argue that benzos should be viewed as a first-line treatment for anxiety, including in the form of long-term treatment for chronic and relapsing anxiety, such as GAD (26, 318, 329; see also, 28, 88). Even authors who deny that benzos are a first-line anxiety treatment for GAD nevertheless defend the value of long-term use in other anxiety disorders, and express concern that overemphasis on its dangers may lead to the selection of less effective treatments (27, 73-74).

There is currently an impasse around what some have dubbed the “paradox of long-term benzodiazepine treatment” (28, 29; cf. 89). On one hand, many people misuse and overdose on benzos. Furthermore, there is evidence that some anxiety patients become severely dependent on benzos and suffer from debilitating withdrawals and other adverse consequences from long-term use. On the other hand, morbidity problems notwithstanding, epidemiological data show the general safety of long-term benzo prescribing. A resolution to the paradox is not forthcoming. Silberman and colleagues suggest that the bad outcomes of long-term benzo prescribing occur when patients have “maladaptive personality traits” or providers prescribe benzos for “dysphoria secondary to life stressors and difficulties,” and suggest that misuse and dependence will be largely eliminated when benzos are prescribed as indicated to patients who are not generally prone to drug misuse (e1). These authors are probably right that some patients are at higher risk of becoming dependent in long-term benzo use, and that screening for risk factors will reduce its worst outcomes. But the risk factors they propose are speculative and require further study. There are several gaps in our knowledge about long-term benzo prescription and use that impede an evidence-based assessment of their safety and efficacy for various anxiety disorders. (1) While long-term benzo use is relatively safe on the whole, there is virtually no research on its long-term efficacy for anxiety disorders, especially in comparison with alternatives like SSRI and SNRI antidepressants. Benzos may be more or less effective anxiety treatments than alternatives in the long run, and better understanding here would affect the overall risk-benefit analysis around long-term benzo prescribing. (2) There is also scant data on the safety or efficacy of long-term benzo use for acute anxiety (as occurs in specific phobias, social anxiety disorder, and panic disorder) as opposed to the chronic anxiety found in GAD and post-traumatic stress disorder (PTSD). As fast-acting, relatively potent anxiolytics, benzos seem better suited to the treatment of acute rather than chronic anxiety; but there is insufficient evidence to settle the question. (Notably, benzos may slow recovery in PTSD and are often recommended against for this application; see, for instance 90.) (3) While certain factors have been associated with benzo misuse—such as youth, and the presence of other substance use disorders—we know almost nothing about what puts some patients at risk for severe dependence and withdrawals at normal prescription doses. Nor do we know (4) what percentage of long-term benzo prescribees become severely dependent following use as-prescribed, and whether this primarily occurs at higher or more potent dosages, or also at lower, milder dosages. Finally, (5) some apparent harms of long-term benzo use are not well understood. The growing community of people who have been injured by long-term benzo prescription and use cite “benzodiazepine-induced neurological dysfunction” (BIND) as a primary danger of long-term benzo use. BIND is not widely discussed in psychiatric benzo research, likely because past concerns about benzo-caused dementia have largely been ameliorated (62, 63). But BIND does appear to be a significant risk of long-term benzo use, and efforts to build scientific BIND literature are underway. A survey of current and former benzo users (N=1,207) revealed over 20 widely overlooked symptoms of benzo use—including lethargy, distractedness and memory loss—which respondents report to interrupt normal functioning for a year or more (56). These authors rightly call for further research into the risk factors for these symptoms, but further research is also required to determine their prevalence, and the validity of the BIND category as a unified syndrome.

## 4 The as-needed black box

Long- and short-term benzo use are both normally defined as durations of daily use: short-term benzo use is the use of a benzo once or more per day for up to four weeks, while long-term benzo use is use at least once per day for more than four weeks. Most studies on the safety and efficacy of benzos concern these two forms of daily use, and the greatest risks of regular benzo use—tolerance, dependence, and withdrawal—are usually attributed to long-term use. This is the main reason for the controversy around long-term benzo prescription, with which the benzo discourse is currently preoccupied.

The focus on daily benzo prescription and use leaves two vitally important and closely related categories overlooked: as-needed prescription and use, and intermittent use. As-needed use (also known as *pro re nata* or PRN) is the use of a treatment in response to acute symptoms, and contrasts with regularly scheduled treatment. Intermittent use is use that occurs in episodes, separated by periods of non-use. A patient who experiences anxiety daily might take their medication strictly as-needed, but nevertheless on a daily basis—not because they are scheduled to, but in direct response to acute symptoms. This is more likely in chronic anxiety conditions like GAD. On the other hand, a patient who does not experience anxiety all the time, but in response to non-ubiquitous cues or triggers, will normally take their medication intermittently, as-needed. This can look like days or weeks of use once or more daily, which then ceases when the anxiety subsides, and is more likely in conditions like social anxiety disorder, specific phobias, and panic disorder.

As fast-acting anxiolytics, benzos are particularly well-suited to the treatment of acute anxiety. They are, therefore, particularly well-suited to as-needed use, and this appears to be how they are most commonly prescribed (see below: 37, 41). Naturally then, we would like to be able to speak to the safety and efficacy of as-needed benzo prescribing for particular anxiety disorders. If we assume that long-term benzo use is the dangerous form, we might hypothesize that the safety of as-needed use depends on whether it is intermittent, and whether episodes of use exceed short-term use (again, widely considered safe). We might also expect that benzos will generally retain anxiolytic efficacy even when prescribed for as-needed use in the long term, since they tend to retain this efficacy in long-term (daily) use. Given that some anxiety disorders involve chronic anxiety while others do not, we can reasonably hypothesize that as-needed prescribing will be likely to produce long-term daily use in some conditions, and unlikely to do so in others—perhaps recommending benzos as-needed differentially across various anxiety disorders. Then again, it seems that long-term daily benzo use is relatively safe and effective for the majority of anxiety patients, and specifically those who do not have certain (largely unknown) risk factors. And it may be that intermittent as-needed benzo use has its own distinct advantages and risks, pertaining, just for instance, to dependence potential or BIND.

Unfortunately, we know very little about either as-needed prescription and use, or intermittent benzo use. The view that benzos are most often prescribed for as-needed rather than regular scheduled use is supported by a small selection of studies showing that most benzo prescriptions are issued by general or family physicians rather than psychiatrists (41), and that most benzo prescriptions by generalists are for as-needed use (while psychiatrists prefer regularly scheduled use, but nevertheless prescribe benzos for as-needed use over half the time) (37, 61-62). This is the result we should expect, given that there are more alternatives to benzos for regular scheduled use (e.g. SSRIs) than for as-needed use. A couple of studies have found that patients who are prescribed benzos for various indications gradually move towards as-needed self-administration (91, 92), while others show a high general prevalence of as-needed use (37, 60). Only a handful of studies have addressed the efficacy of as-needed use for anxiety disorders, with one finding it effectively anxiolytic (93), and others finding lower reduction of attention to threat that CBT (94, 95). The safety of as-needed benzo use is all but unstudied, with some authors expressing concern about increased dependence potential relative to regular use on the basis of research on narcotic analgesics (37, 67-68). Indeed, the one area in which as-needed benzo prescribing is well studied is psychiatric hospitalization, viewed as a site for deprescribing (31–36). These studies tell us nothing about how providers prescribe to patients for as-needed self-administration, how patients self-administer, or the safety and efficacy of outpatient, as-needed use.

The dearth of information about as-needed and intermittent prescription and use of benzos is due to the fact that, with a couple exceptions, studies on benzo safety and efficacy only distinguish short-term and long-term regular use (cf. 37–40). This design choice significantly limits applicability in clinical practice. It has several probable rationales. For one, as-needed and intermittent use require higher resolution measurement to track, making them more difficult to study. That is, they require the tracking of every dose, whenever it happens to occur, though doses occur on an irregular basis. This is much less practicable than relying on the duration of a term of prescription, which can usually be found in medical records. Relatedly, the assumption that long-term prescription implies daily use errs on the side of caution. It is safer to assume that long-term prescription recipients use daily, than that they do not. Finally, it is technically possible to analyze all intermittent or as-needed use as either long-term or short-term; regardless of whether use is as-needed or intermittent, any given period of use either involves more than four weeks of daily use, or it does not. Of these rationales, the first is clearly legitimate, the others less so. The major gaps in benzo research demand the study of as-needed and intermittent prescription and use, and to the extent that there are technical or logistical limitations to this capacity, it is crucial that they be addressed.

## 5 The urgent need for comprehensive evidence-based best practices

Several coinciding societal factors make it more important than ever that medical providers can “walk the highwire” (27) over overprescription and underprescription, particularly in the U.S. On one hand, mental health is getting worse in the U.S. and abroad, to include increases in the prevalence of anxiety disorders. According to a recent U.S. National Health Statistics Report, the percentage of adults with symptoms of anxiety or depression increased significantly between 2019 and 2022, from 15.9 to 18.2 percent and from 18.5 to 21.4 percent, respectively (45). American youth have seen especially remarkable increases in anxiety symptoms for over a decade (96), with the U.S. Centers for Disease Control and Prevention (46) proclaiming that “[y]outh in the U.S. are experiencing a mental health crisis.” This mental health crisis is not a distinctly American problem; one study estimates that rates of anxiety among adults has increased by more than 55 percent globally between 1990 and 2019, with anxiety disorder metrics showing a continuous increase in prevalence, incidence, and DALY rates (44). On the other hand, access to mental and behavioral healthcare is limited. The U.S. Health Resources and Services Administration’s (HRSA) First Quarter of Fiscal Year 2025 Designated HPSA Quarterly Summary estimates that over 122 million Americans live in a designated mental health professional shortage area (HPSA)—with only 26.44 percent of need met across all those mental health HPSAs together, and over 6,200 providers required to meet the needs of those areas (97). To make matters worse, non-pharmaceutical treatments for anxiety that are considered safer and more effective than benzos—first and foremost, CBT—are largely inaccessible. Drawing on a range of therapist survey studies, Woltizki-Taylor and colleagues argue that “access to and receipt of evidence-based treatments for mental health disorders, and exposure-based treatments for anxiety disorder in particular [i.e. CBT], remain shockingly low. Indeed, despite the existence of highly effective treatments for anxiety disorders, most individuals with anxiety disorder do not receive them” (47, 899). Further dissemination of cognitive-behavioral therapies, including novel methods of delivery, is urgently needed (48).

Given the already limited access to mental healthcare in the U.S. and many other places, the emphasis on benzo deprescribing that currently prevails in the health sciences can be an obstacle to people seeking care for anxiety disorders. Some primary care and psychiatry providers have come to doubt that benzos are even therapeutic and adopt a roundly anti-benzo policy (98, 3). These providers run the risk of denying needed mental healthcare to patients who lack access to alternatives, or even a second opinion. Needless to say, overprescribing is also a serious risk, especially prescribing to patients who do not have an indication for benzo pharmacotherapy and who have risk factors for misuse. This dilemma that providers face—to err toward more liberal or more restrictive prescribing practices—is precisely why evidence-based best practices for benzo prescribing are so urgently needed. Providers must be able to prescribe benzos with confidence when they are the best available treatment, and to provide alternatives, or connections to alternatives, when not. As I argue above, a great deal more research must occur before authoritative best practices can be formulated.

## 6 How to prescribe, for now

The best available evidence does not show benzos to be extremely dangerous drugs that must generally be avoided. The primary dangers of benzos are dependence and withdrawal, which typically occur in long-term daily use. While long-term daily use does carry these risks, it appears to be safe and effective for many (though not all) chronic anxiety patients, who rarely develop tolerance or escalate dosage to enhance efficacy. The risk and associated factors relating to other effects of long-term benzo use, such as BIND, are not well understood, but are probably not a risk for most candidates. Nonetheless, since alternatives like SSRIs and buspirone are roughly as effective for anxiety as long-term benzo use—and arguably, safer—providers should carefully assess whether long-term daily benzo use is the best option for a patient with chronic anxiety.

It is important that providers appreciate the difference between (a) long-term daily benzo use and (b) long-term prescription for as-needed, intermittent benzo use. Patients who suffer from non-daily acute anxiety due to conditions like specific phobias, social anxiety disorder, and panic disorder appear to be particularly good candidates for treatment with as-needed benzo prescriptions. While more research on intermittent and outpatient PRN benzo use is urgently needed, at present, there is little reason to think this mode of prescription is more dangerous or less effective than for short-term use. The primary difference is that in long-term prescription for intermittent, as-needed use, it is sometimes possible for patients to use every day for long periods of time. With this comes the risk of dependence, which is lower when the total duration of the prescription is one month or less. But crucially, the risk of long-term prescription for as-needed use is easily reduced by prescribing in lower quantities. Available evidence suggests that fifteen or twenty doses of a low-potency benzo per month is unlikely to give rise to dependence, even after several years, since at those quantities it is impossible (without stockpiling) to take one or more doses per day for more than two or three weeks. While the most problematic patterns of use at those quantities (e.g. three or four doses per day for a week) would be worrisome and would call for alternative treatment, in all likelihood, the vast majority of patients with acute situational anxiety will not use an as-needed prescription in this way against medical advice. (Again, there is evidence of high rates of patient compliance with benzo prescriptions.) In short, then, providers have reason to feel secure in prescribing low quantities of benzos for as-needed, intermittent use to patients with a clear indication, i.e. a diagnosed anxiety disorder involving acute situational anxiety, in the absence of counterindications. Often times, such prescriptions can be safely managed even for long periods.

Perhaps more than any other factor, proper medication management is vital to safe and effective benzo treatment. Regular follow-up appointments, patient outreach, and communication via online portals are all valid methods for tracking patient behavior and gauging the success of a course of treatment. Providers must ensure that patients are not using their medication in excess, that they are not escalating dosage, and that they are not exhibiting serious side-effects or interdose withdrawals. One of the best reasons for conservatism around benzo prescribing is difficulty providing active medication management. But it is incumbent on both primary care providers and specialists to find adequate means of managing patient prescriptions with serious risks, which includes not just benzos, but a vast array of valuable pharmaceuticals. Again, anxiety patients often have limited access to mental or behavioral healthcare. If they cannot get needed care from the providers to whom they do have access, they may not get treatment at all, even when they have profoundly disabling anxiety disorders. This can lead to harmful self-medication with alcohol or other dangerous drugs.

The accessibility of non-pharmaceutical alternatives is also an important consideration when evaluating patients for any form of benzo pharmacotherapy. Exposure-based CBT in particular appears to be safer and more effective than benzos and comparable pharmaceutical interventions for both acute and chronic anxiety. As such, cognitive-behavioral interventions are to be preferred over benzos when they are readily accessible to a patient. However, CBT is not widely available, and when a provider cannot make a warm hand-off to a CBT specialist, the accessibility of CBT should not be presumed. Given how debilitating anxiety disorders can be, benzo pharmacotherapy should be seriously considered in the absence of accessible cognitive-behavioral alternatives. The superiority of CBT is not a reason to withhold benzo pharmacotherapy when the superior modality is not readily available. The increasingly endorsed view that benzos are not a first-line treatment for anxiety can obscure the fact that they are not merely adjunctive or last-resort treatments for anxiety disorders.

## 7 Conclusion

Attitudes towards benzos have fluctuated since soon after their introduction in 1960. The last decade or so has seen a wave of concern about their safety, and how best to use them in the treatment of anxiety disorders. Recent studies have correlated rising rates of benzo prescription and rising rates of benzo-related overdose mortality, and there is growing awareness of enduring negative effects from long-term benzo use, even when taken as prescribed. Benzos are habit-forming, and severe dependence involves excruciating, medically dangerous withdrawal. As central nervous system depressants, they are particularly dangerous when taken in combination with other depressants like opioids, with which they are often co-prescribed. To be sure, attention to the risks of benzo misprescription and misuse is called for.

While benzo pharmacotherapy carries significant risk, it is also an invaluable strategy for the management of anxiety disorders, particularly acute anxiety. The therapeutic value of benzos has been obscured by the focus on the associated risks, a fact reflected in the content and tone of public health guidance (e.g. FDA boxed warnings) and recent best practices literature. Due to significant gaps in the research, comprehensive evidence-based guidance about how to prescribe benzos for anxiety is not forthcoming. Particularly alarming is the absence of research on the relative safety and efficacy of as-needed and intermittent benzo use. As fast-acting anxiolytics, benzos are particularly well-suited to the management of acute anxiety, and available evidence suggests that benzos are more frequently prescribed for as-needed use than long-term daily use. Furthermore, an international mental health crisis and strained mental and behavioral healthcare systems create pressure to deploy what resources are readily available. Research into the safety and efficacy of as-needed and intermittent benzo use for anxiety is therefore urgently needed. That said, we have no scientific reason to suspect that as-needed or intermittent benzo use is generally dangerous; and insofar as it is equivalent to short-term use, we have good reason to think it is highly safe and effective for various anxiety disorders. Underprescription of benzos for as-needed use is a likely consequence of current messaging around benzo prescription and use and would constitute a significant weakness in already-strained healthcare systems. Crucially, the view that benzos are never first-line interventions for anxiety due to high misuse potential is not evidence-based. Our best science indicates that benzos have relatively low potential for misuse and are relatively safe and effective pharmaceutical interventions for anxiety. In this scientifically non-ideal situation, and given mental healthcare shortages, providers have a responsibility to write and manage benzo prescriptions for as-needed use when better anxiety treatments are not available.
